# Investigation on the Effect of a Pre-Center Drill Hole and Tool Material on Thrust Force, Surface Roughness, and Cylindricity in the Drilling of Al7075

**DOI:** 10.3390/ma11010140

**Published:** 2018-01-16

**Authors:** Amir Hossein Ghasemi, Amir Mahyar Khorasani, Ian Gibson

**Affiliations:** 1 Department of Manufacturing and Production, University of Kashan, Qotb-e Ravandi Blvd., Kashan 8731753153, Iran; ah.ghasemi@grad.kashanu.ac.ir; 2 School of Engineering, Faculty of Science, Engineering and Built Environment, Deakin University, Victoria 3216, Australia; Ian.gibson@deakin.edu.au

**Keywords:** drilling, thrust force, pre-center drill hole, surface roughness

## Abstract

Drilling is one of the most useful metal cutting processes and is used in various applications, such as aerospace, electronics, and automotive. In traditional drilling methods, the thrust force, torque, tolerance, and tribology (surface roughness) are related to the cutting condition and tool geometry. In this paper, the effects of a pre-center drill hole, tool material, and drilling strategy (including continuous and non-continuous feed) on thrust force, surface roughness, and dimensional accuracy (cylindricity) have been investigated. The results show that using pre-center drill holes leads to a reduction of the engagement force and an improvement in the surface quality and cylindricity. Non-continuous drilling reduces the average thrust force and cylindricity value, and High Speed Steels HSS-Mo (high steel speed + 5–8% Mo) reduces the maximum quantity of cutting forces. Moreover, cylindricity is directly related to cutting temperature and is improved by using a non-continuous drilling strategy.

## 1. Introduction

Drilling is one of the popular machining methods and is used in most assembly processes. Various aspects of drilling have been investigated, such as process modelling, tool material, cutting force, and surface roughness [1,2,3,4,5].

Klocke and Krieg studied various properties of coated drills. They found tool coating life is associated with workpiece materials due to the formation of a built-up edge on cutting edges, and this problem can be solved by using appropriate cutting fluids. The affinity of coated layers and workpiece materials is found to be the main reason for a reduction in tool life [6]. Kelly and Cotterell compared the effects of traditional and minimum quantity lubricant (MQL) to analyse the effect of lubrication on surface roughness, thrust force, and machining torque. The results proved that the MQL nozzle, workpiece material, and MQL particle size are significant to the generation of thrust force and surface roughness [7]. Tsao and Hocheng investigated the effects of cutting-edge length and tool diameter on composite delamination during the drilling of fibre-reinforced composites. The outcomes of this research illustrated that increasing the cutting-edge length and feed rate is a direct function of delamination. Furthermore, the value of thrust force is related to the hole depth. They also presented analytical models with acceptable accuracy compared to the experimental results [8]. Paro et al., studied the drilling of X2 Cr Ni 19 11 stainless steel by using TiN- and TiCN-coated tools. They investigated the effect of feed rate and cutting speed on thrust force and tool life, and found that feed rate is the most influential factor on chip formation [9]. Bakkal et al. studied bulk metallic glass (BMG) drilling and analysed the effect of the cutting condition on thrust force, torque, and tool wear. They found that WC-Co (Tungsten carbide + Cobalte) is an acceptable tool for drilling BMG, and by increasing the cutting speed and decreasing the feed rate the mentioned results were improved. However, drilling at a higher speed causes faster and more tool wear [10]. Astakhov and Galitsky studied tool life on malleable cast iron by using single-edge drill bits. The effect of different tool angles and feed rates was studied and optimized [11]. Bagci and Ozcelik studied the effect of depth, feed rate, and cutting speed on drilling temperature and thrust force in Al7075 by using TiN/TiAlN-coated carbide drills. The result showed that drilling depth is an effective parameter on machining temperature followed by cutting speed and feed rate [12]. Ke et al. investigated chip formation during deep hole drilling and proposed an analytical model to predict the machining force during the drilling process [13]. Using a core drill leads to increased feed without increasing delamination in the drilling of composites, and the efficiency of the process improves by optimization in tool design. Also, in the machining of composites, using coated HSS drills leads to an enhancement of surface quality [14,15,16]. Olovsjo et al. studied grain size and hardness effects in the drilling of Inconel 718 by a cemented carbide tool and observed that the hardness and grain size affect the deformation layer of workpieces [17]. Using design of experiment methods, such as Taguchi and response surface, showed that feed rate and cutting speed are the most influential parameters on the drilling of steels. Different machining methods, such as ultrasonic drilling, and tool selection leads to better surface quality and drilling of soft materials, such as Al, and helical milling increases fatigue life [18,19,20]. The effect of pre-hole drilling on Al 6061-7075 with uncoated HSS drills showed that pre-holes reduce cutting force and improve surface roughness and chip generation [21].

P. Nieslony et al. studied the effects of tool coating in the drilling of explosively clad Ti–steel plates and found that tool material affects drilling force, torque, and surface morphology [22]. They also compared the surface morphology produced by WC-Co- and TiAlN-coated tools in different machining zones and found that drilling with carbide tools can achieve better surface quality [23]. M. Percin et al. [24] studied the effect of machining parameters and machining conditions on the force, torque, surface roughness, and tool wear in the micro-drilling of Ti-6Al-4V and found that both the machining parameters and cutting condition have an effect on drilling force and torque and that the built-up edge (BUE) is highly related to the tool wear mechanism. F. Berzosa et al. [25] studied tool selection and cutting condition in the drilling of Magnesium UNSM11917. They found that minimum quantity lubricant has a significant effect on reducing surface roughness and that cutting speed is the most effective parameter that affects surface roughness. B. Vakili Azghandi et al. [26] studied the relation between ultrasonic vibration-assisted drilling and cutting force, and found that using ultrasonic vibration causes a decrease in the average force of drilling. Suman Chatterjee et al. studied the simulation and optimization of cutting force and torque in the drilling of Ti-6Al-4V alloy using Deformed 3D software, and they report optimal values of cutting speed and feed rate to achieve the lowest drilling force [27].

However, there appears to be a lack of research into the use of different cutters, drilling strategies, and pre-center hole drilling on cutting force, surface quality, and cylindricity.

Tool selection and tolerances are the most important factors in the machining of Al7075. An accurate tool selection and low dimensional deviations improves industrial parts in terms of production cost, time, and quality. This paper investigates the effects of tool material, a pre-center drill hole, and drilling strategy on the thrust force in and surface roughness of drilled holes. A full factorial design of experiment (DOE) has been selected to achieve higher accuracy.

## 2. Material and Methods

**Figure 1:**
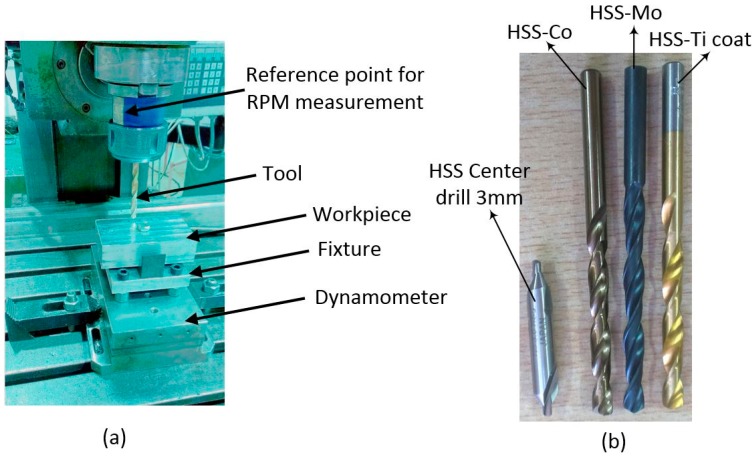
(**a**) Drilling setup; (**b**) Cutting tools. RPM: revolutions per minute.

Our experimental procedure includes drilling followed by the measurement of force, surface roughness, and cylindricity. A computer numerical control (CNC) milling machine with a hydraulic transfer system was used as shown in Figure 1a.

The thrust force of a drilling operation was measured using a Kistler 5070 dynamometer, and to avoid aliasing noise a 2 kHz sampling frequency was selected, which is approximately ten times greater than the spindle speed. The hole surface roughness of Al 7075 was measured by the Mahr PS1 surface roughness tester (Elcometer, Singapore) in eight different positions. (Table 1 shows the chemical composition of Al 7075 alloy).

Three different drills were used for the machining operations, comprising HSS-Ti-coated, HSS-8% Co, and HSS-Mo, all with a 7 mm diameter (Figure 1b). Table 2 and Table 3 show the geometric and machining parameters, respectively.

To obtain higher accuracy, a full factorial DOE for two factors in three levels has been used [28,29].

Three different drilling strategies—continuous, non-continuous with 1 mm reciprocation movement (G82), and non-continuous with full reciprocating movement (G83)—were used in this experiment. The effect of drilling with and without the pre-center drill holes was also studied in all experiments. Tool overhang was 73.52 ± 0.02 mm and was controlled before each machining operation. The orthogonality of the cutting tool and the workpiece was monitored by a dial indicator after each tool change and ±5 µm was obtained, which was considered acceptable. To have a consistent temperature throughout the experiments, between each two tests the machine was turned off for 30 min. After each test, the cutting edge was monitored by a Visual Measuring Machine (VMM, Yihui, Guangdong, China) to check tool wear and maintain consistency in the experiments.

**Figure 2:**
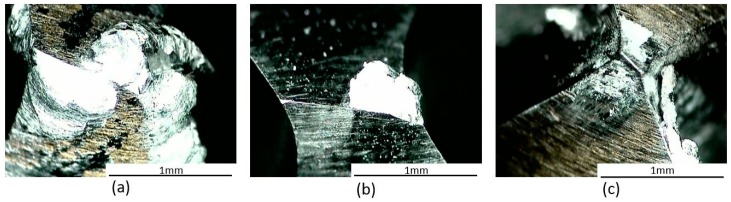
Worn drills (**a**) HSS-Ti; (**b**) HSS-Mo; (**c**) HSS-Co.

Tools with a built-up edge and cracking are known as worn tools. However, just the built-up edge was observed in these experiments. Figure 2 shows the built-up edge for different drill bits.

Dry machining has been carried out, and after drilling, eight points at 5.6 mm length intervals from the inside of each hole were selected. The average value of these points was reported as each hole′s surface roughness.

## 3. Result and Discussion

### 3.1. Thrust Force and Surface Roughness

**Figure 3:**
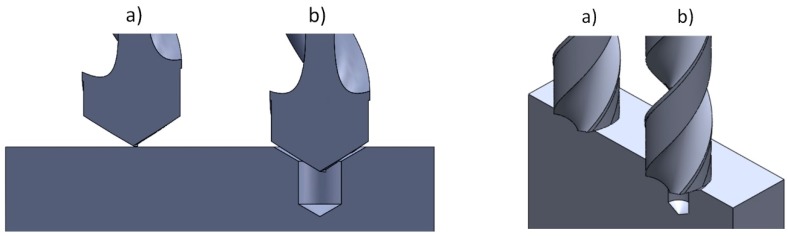
Drill position (**a**) without a pre-center drill hole; (**b**) with a pre-center drill hole.

Table 4 shows the design of the experiment. The engagement status of the drill and the workpiece for both with and without a pre-center drill hole is shown in Figure 3.

According to Figure 3, it is obvious that in the workpiece with the pre-center drill hole, engagement starts with the initial cutting edge on the cutter. Without using a pre-center drill hole, the initial point of engagement is seated on the chisel edge. The cutter may therefore slip on the workpiece and consequently angular drill holes may be generated. In addition, tool break may be observed in the case of using brittle cutters (such as WC).

**Figure 4:**
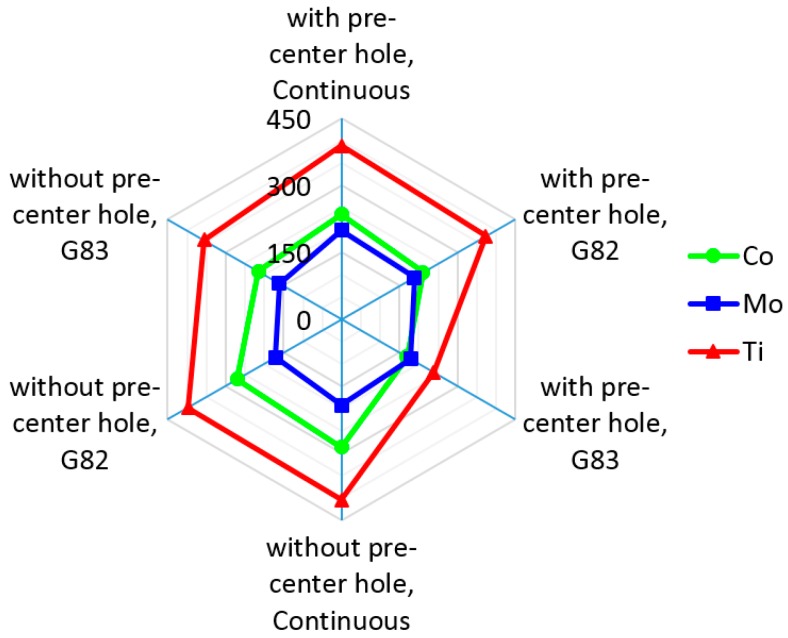
Cutting thrust force results for different cutters and strategies.

Figure 4 illustrates that HSS-Ti coated has the highest thrust force followed by HSS-Co and HSS-Mo. This trend was also observed in all cutting strategies. The differences between force values are associated with the difference in friction coefficient and edge (sharpness, the quality of coating, coating material, and cutting-edge angles) [30,31,32]. In a drilling operation, cutting-edge preparation is more important than the friction coefficient [33,34,35]. The higher cutting force for HSS-Ti-coated tools is also related to the coating material on the cutter. Ti has low thermal conductivity and higher temperature in machining when using a Ti cutter, which is reported in the literature [36,37]. Moreover, using a Ti cutter generates higher temperature and with respect to the high affinity of this alloy to chemically react with most of the materials at higher temperatures tool wear was observed in the cutting edges and consequently higher cutting forces were also observed [37,38,39].

**Figure 5:**
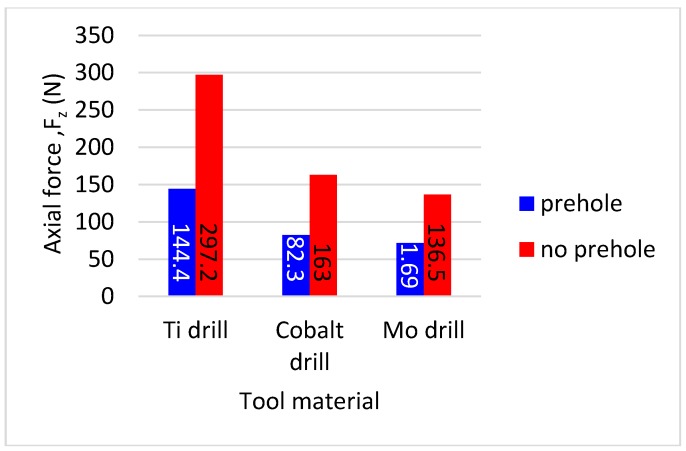
Comparison of a pre-center drill hole and no pre-center drill hole on drilling engagement force.

Reducing the thrust force leads to a reduction in negative effects on the tool and the workpiece as well as decreasing the temperature of the drilling operation. This is related to the direct impact of the thrust and friction forces. Therefore, in the case of lower thrust forces, no significant effect was observed for using various drilling strategies on the value of the cutting force. A pre-center drill hole does not change the maximum value of the thrust force, but reduces the engagement force (the average force from the first contact point on the tool and workpiece to reach the maximum (constant) thrust force) and subsequently buckling phenomena. In static mode, the tool is assumed to be a single head-free beam with full support at the end and with axial load. Therefore, loading in the cross-section of the cutter results in a buckling effect [40,41,42,43,44,45]. The comparison between engagement forces on the hole with and without a pre-center drill is demonstrated in Figure 5. This figure shows that using a pre-center drill reduces engagement force, which is supported by the literature [21].

**Figure 6:**
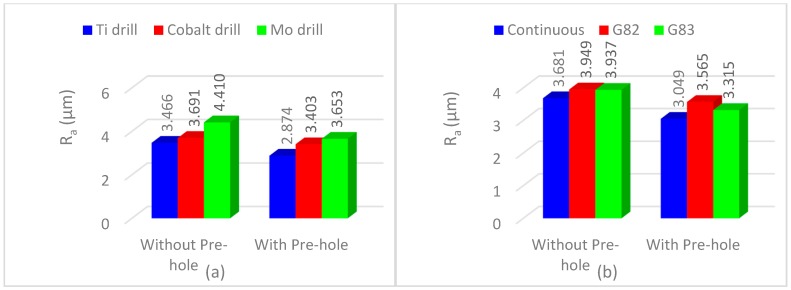

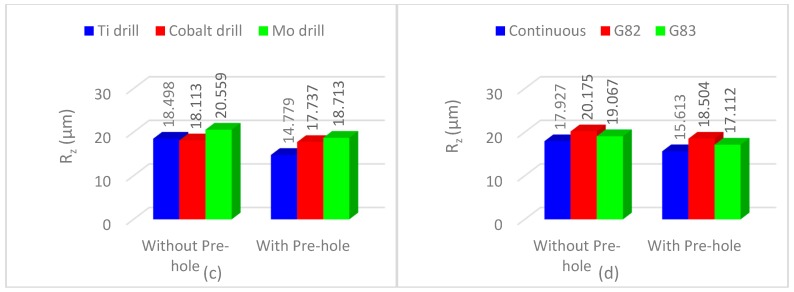
The effect of using different cutters and strategies on (**a**,**b**) average roughness and (**c**,**d**) mean roughness depth.

Figure 6 shows the effect of a pre-center hole and the use of different cutters on the hole’s surface roughness. According to Figure 6a,c, HSS-Ti-coated tools generated the best surface quality followed by HSS-Co and HSS-Mo. This trend is associated with the value of the damping ratio [46,47]. The HSS-Mo cutter has the lowest cross-section area and mass, which results in higher buckling (according to Equation (1)) and vibration and a lower damping frequency than the HSS-Ti-coated cutter. Therefore, using HSS-Mo generates more vibrations and higher roughness values. According to these results, choosing HSS-Ti-coated drills and the continuous strategy leads to the generation of better surface roughness. Figure 6a,c shows that using a pre-center hole produced better surface quality that is mainly related to a reduction in the engagement force and a lower buckling effect [40,41,42,43,44,45].

Figure 6b,d illustrates that using the continuous strategy generated the best surface quality followed by G83 and G82. In non-continuous strategies, the cutting edges have negative effects on the surface quality in subsequent drilling passes. Therefore, a small cutting process occurs in subsequent passes and generates rougher surfaces. This phenomenon occurs even when the spindle or drill bit has a small imbalance.

Maximum buckling and tool deformation can be calculated by Equation (1).

(1) $$y_{\max}=e\left[\sec\left(\sqrt{\frac{p}{EI}}\cdot \frac{L}{2}\right)-1\right]$$

where, *P* is the thrust force, *L* is the tool length, *E* is Young′s modulus, and *I* is the second moment of inertia with respect to the drill’s Z axis.

Due to the complex structure and geometry of the tool, calculation of the exact value of buckling is complicated. As shown in Equation (1), increasing the thrust force leads to an increase in the value of tool buckling (*y*_max_).

It should be considered that, on the first contact between the drill and the workpiece, a slipping effect due to vertical offset may occur. When using a pre-center drill, this problem is solved because of the smaller cutter length and larger moment of inertia. This problem is more common in thinner drills.

The results showed that HSS-Mo presented the best machining conditions for the reduction of cutting force. Using drills with tapers leads to the generation of better roughness and lower dimensional deviations [48].

### 3.2. Geometrical Dimension and Tolerance

**Figure 7:**
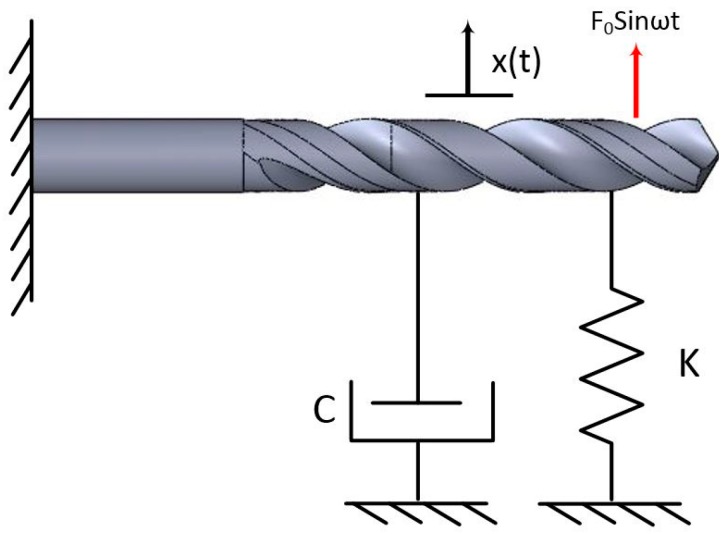
Mass-spring system for the cutting tool.

Cylindricity increased by using a pre-center drill hole for all experiments that are associated with reducing engagement force (Figure 5). According to Equation (1), forces P and L reduce and the value of I increases, therefore lower buckling and higher cylindricity were observed [40,41,42,43,44,45]. The best cylindricity was obtained when using HSS-Co tools, followed by HSS-Mo and HSS-Ti-coated tools. Figure 4 shows that Ti has the highest cutting force and buckling values and therefore the lowest cylindricity is related to higher vibration. In this experiment, due to using Al as a workpiece and the high hardness of the cutters comprising HSS-Ti: 856.4 Hv, HSS-Co: 910 Hv, and HSS-Mo: 667 Hv, no torsional vibration was observed on the workpiece and it is neglected. Vibrations with external loads are obtained from the following ordinary differential equation.

(2) $$M\ddot{x}\left(t\right)+C\dot{x}\left(t\right)+Kx\left(t\right)=F_{0}Sin\omega t⋝\;\ddot{x}+2\xi \omega_{n}\dot{x}+\omega_{n}^{2}x=F_{0}\;Sin\omega t$$

where M is the cutter’s mass, C is the damping coefficient, K is the stiffness ratio, *F*_0_ is the external force, and $\ddot{x}\left(t\right),\;\dot{x}\left(t\right),\;\mathrm{and}\;x\left(t\right)$ are the acceleration, velocity, and displacement, respectively. Figure 7 shows a mass-spring model for the drill bit used in this experiment. $\omega_{n}$ is the natural frequency and *ξ* is the damping ratio that is calculated from Equation (3).

(3) $$\xi =\frac{C}{C_{cr}},\;\xi =\frac{C}{2M\omega_{n}}$$

where *C_cr_* is the critical damping and Equation (2) has both a general and particular response. By assuming a critically damped position (*ξ* = 1) and solving the second differential equation of motion, the general response is obtained according to Equation (4):

(4) $$x_{g}\left(t\right)=Ae^{-\xi \omega_{n}t}\sin\left(\omega_{d}t-\psi \right)$$

where *ψ* is the initial phase difference for the general response and *A* is the amplitude of the vibrations. The particular response of Equation (2) is:

(5) $$x_{p}\left(t\right)=x_{0}\sin\left(\omega_{d}t-\phi \right)$$

where *X*_0_ is the initial displacement and ϕ is the initial phase difference for the particular response, which is obtained from Equations (6a) and (6b).

(6a) $$x_{0}=\frac{F_{0}}{\sqrt{{\left(K-M\omega^{2}\right)}^{2}+{\left(C\omega \right)}^{2}}}$$

(6b) $$\phi =\tan^{-1}\frac{C\omega }{K-M\omega^{2}}$$

Based on the following relations, Equations (6a) and (6b) are converted to a dimensionless quantity:

(7a) $$\frac{Kx_{0}}{F_{0}}=\frac{1}{\sqrt{{\left[1-{\left(\frac{\omega }{\omega_{n}}\right)}^{2}\right]}^{2}+{\left[2\xi \frac{\omega }{\omega_{n}}\right]}^{2}}}$$

(7b) $$\phi =\tan^{-1}\frac{2\xi \frac{\omega }{\omega_{n}}}{1-{\left(\frac{\omega }{\omega_{n}}\right)}^{2}}.$$

In our experiments, the initial conditions are, *x*(0) = 0 and $\dot{x}\left(0\right)=0$, so by determining Equations (5)–(7), the particular response is obtained from Equation (8).

(8) $$x\left(t\right)≅\frac{1}{2\xi }\frac{F_{0}}{K}\left[e^{-\xi \omega_{n}t}\left(\frac{\xi }{\sqrt{1-\xi^{2}}}sin\omega_{d}t+cos\omega_{d}t\right)-cos\omega_{n}t\right]$$

In this experiment, due to using cutting tools with a high value of hardness $\frac{\xi }{\sqrt{1-\xi^{2}}}sin\omega_{d}t\approx 0$ and assuming $\omega_{n}=\omega_{d}$ and $\phi =\frac{\pi }{2}$, the particular response is:

(9) $$x\left(t\right)≅\frac{1}{2\xi }\frac{F_{0}}{K}\left(e^{-\xi \omega_{n}t}-1\right)cos\omega_{n}t.$$

According to Equation (9), the amplitude of the vibrations is a function of force, and when using HSS-Ti tools a higher force was captured; therefore, by increasing *F*_0_ in the right-hand side of Equation (9), the value of *x*(t) will increase and cylindricity will decrease. This tool is heavier than HSS-Mo; however, due to the higher effect of the cutting force on the vibration compared to the impact of the cutter weight, HSS-Ti generates poorer cylindricity.

**Figure 8:**
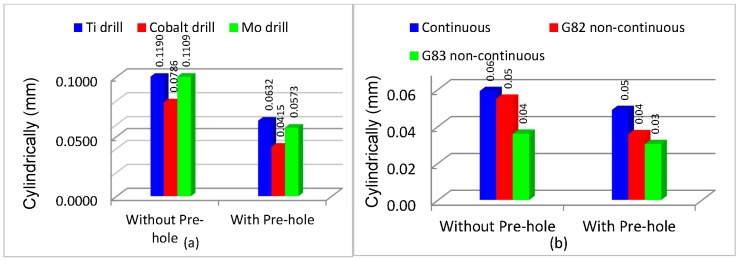
Cylindricity with and without a pre-center drill hole (**a**) for different cutters; (**b**) for various cutting strategies.

**Figure 9:**
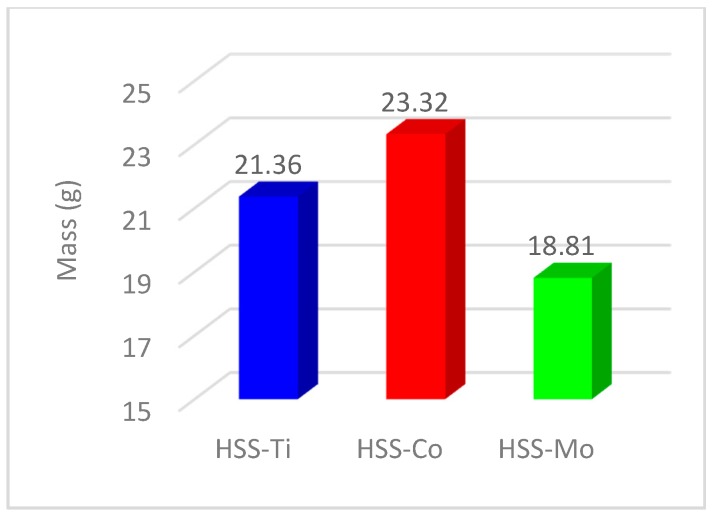
Mass for different cutters.

Figure 8 shows that using the HSS-Mo cutter creates the second best cylindricity value. The reason is associated with the lower mass of this cutter. Figure 9 shows that HSS-Mo is the lightest cutter among all three drills that were used in this experiment. Equation (10) demonstrates that by decreasing the mass, the damping frequency and vibration will increase, which leads to a reduction of dimensional deviations and cylindricity.

Based on the above discussions, the higher overall mass for HSS-Co leads to a reduction in the value of the damping frequency and subsequently improves dimensional deviations and cylindricity and decreases the value of the scrap and the production cost and time.

(10) $$\omega_{d}=\sqrt{\frac{K}{M}-{\left(\frac{C}{2M}\right)}^{2}}$$

G83 was observed to be the best drilling strategy to increase cylindricity followed by the G82 and continuous modes (Figure 8b). The reason can be related to the higher temperature in continuous mode and the better cooling process for the G82 and G83 strategies. Higher temperatures and thermal stresses, notably during drilling, affect dimensional deviations and cylindricity.

Using a pre-center drill greatly improves (about 50%) the dimensional tolerances, and this is also related to the more accurate guide of the drill bit at the starting point. Due to a higher mass and better damping of vibrations during machining, HSS-Co generated better dimensional accuracy compared to HSS-Ti-coated and HSS-Mo.

## 4. The Interaction of Cutting Parameters on Cutting Forces

**Figure 10:**
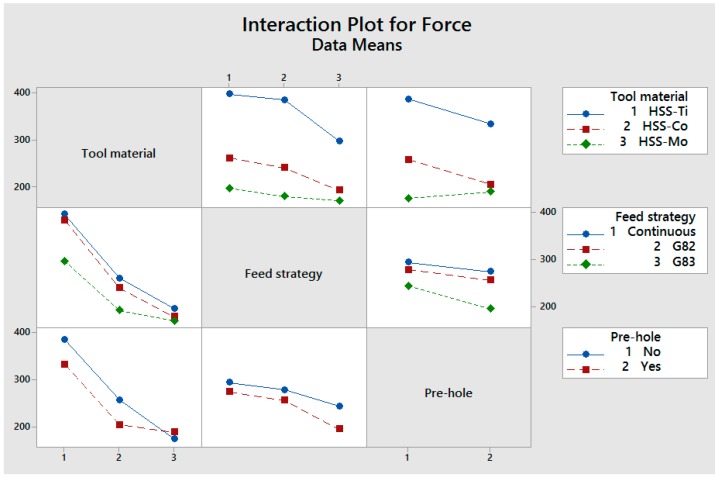
Interaction of cutting conditions versus cutting forces (N).

An interaction plot for cutting conditions versus cutting forces is shown in Figure 10. The HSS-Ti-coated drill generates the highest cutting force, and the reason for this is related to the difference in edge (coating material and cutting edge angles as well as the sharpness and quality of the coating) and friction coefficient [25,26,27]. Another reason for the higher cutting force when using HSS-Ti-coated is associated with the coating material on the cutter. Ti has low thermal conductivity and generates a higher temperature in metal cutting [31,32]. The high affinity of Ti to chemically react with most of the materials leads to an increase in temperature and tool wear and subsequently higher cutting forces [32,33,34]. The interaction of tool material and feed strategy shows that the generated force has a decreasing trend for HSS-Ti, HSS-Co, and HSS-Mo. In G83, less force was observed, which is associated with two possible reasons: (I) a higher cooling time for the drill bit due to its moving back to the reference point; and (II) increasing the depth of the hole leads to a longer movement of the drill bit toward the reference point, increasing the cooling time and subsequently decreasing the cutting force.

The interaction of the feed strategy and tool material shows that for HSS-Ti the cutting force is higher, which is related to higher friction between Ti and Al (the workpiece). Higher generated heat between the cutting tool and the workpiece leads to an increase in the cutting force and tool wear.

The interaction of tool material and pre-hole shows that a 15% and 20% reduction in cutting force were observed when using a pre-hole for HSS-Ti and HSS-Co, respectively. For HSS-Mo, using a pre-hole results in about an 8% increase of cutting force, but this increasing trend is small when using different cutting material. The interaction of a pre-hole and the feed strategy shows that using a pre-hole reduces the cutting force; however, this reduction is negligible when using proper drilling tools (Using HSS-Ti to HSS-Mo leads to a 52% decrease in cutting force and also a 15% reduction was observed for using a pre-hole for HSS-Ti).

The interaction of pre-center drill and tool material shows that using a pre-center drill generates smoother surfaces, which is related to the improved guidance of the drill bit. HSS-Co has the lowest decreasing slope because of the geometry of the drill bit on the chisel edge. HSS-Co has less chisel edge, and this leads to better redirecting of the drill bit toward the hole (even when not using a pre-center drill); however, the use of a pre-center drill further improves the surface roughness.

The interaction of a pre-center drill hole and the feed strategy shows that using a pre-center drill hole prevents any sliding and improves dimensional deviation and surface quality. The continuous feed strategy generated better surface quality compared to G82 and G83. In the reciprocating movement of the drill bit, the end of the cutting edge that engages with workpiece has a negative effect on surface quality, but this problem is compensated for with side-cutting edges. As shown in the results, the differences between the value of roughness when using various cutting strategies are small, with a maximum variation of 15%. More reciprocating movements enhanced the dimensional tolerances that are associated with the role of side-cutting edges in polishing the internal surfaces of the holes.

**Figure 11:**
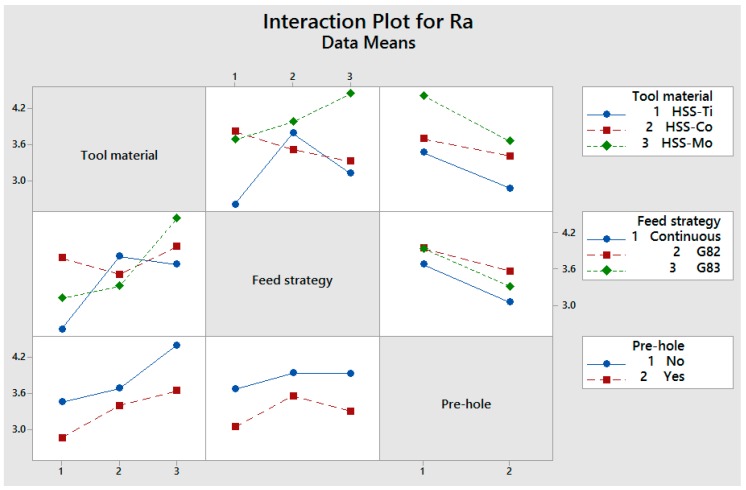
Interaction of cutting conditions versus surface roughness.

As mentioned in previous sections, vibration and the contact portion of side-cutting edges are important factors in the quality of the surface roughness. In addition, roughness increases when the end of the chisel edge is in contact with the workpiece. The interaction of tool material and the feed strategy in Figure 11 shows that the roughness when using HSS-Co is less than when using HSS-Mo and HSS-Ti, which is related to the higher mass and damping. Generally speaking, the differences in the value of roughness are quite small when using different cutting material and the feed strategy.

## 5. Conclusions

In this investigation, the effect of a pre-center drill hole and tool material comprising HSS-Mo, HSS-Co, and HSS-Ti-coated tools on the generated cutting force, surface roughness, and dimensional deviations (cylindricity) has been studied. The results showed that:

- A pre-center drill hole reduces the engagement force, whilst no effect was observed for the average thrust force. This is associated with the difference in material removal rate in each process.
- The best cutting strategy was non-continuous (G83) with full reciprocating movement that reduced the mean value of the thrust force, tool wear, and cylindricity. The effect of cutting edges in subsequent machining passes using this strategy, however, increases surface roughness.
- Using HSS-Mo reduces the maximum value of cutting forces, which is reliant on lower friction coefficients, better surface preparation, and a lower tendency to form built-up edges.
- Cylindricity was found to be a direct function of temperature and using a non-continuous drilling strategy results in better cylindricity and lower dimensional deviations. Indeed, the higher mass for HSS-Co cutters leads to a lower damping frequency and a subsequent reduction in the value of cylindricity.
- Performing these experiments leads to an improvement in dimensional tolerances without interfering with the cost of production.
- By using a pre-center drill hole, manufacturing lead-time and subsequently production cost can be saved.

## Figures and Tables

**Table 1:** Al 7075 chemical composition (weight).

Alloy	Al%	Zn%	Mg%	Cu%	Fe%	Mn%	Si%	Cr%	Ti%
% Weight	Base	5.5	2.5	1.8	0.35	0.3	0.3	0.2	0.1

**Table 2:** Geometric parameters for drill bits.

Parameter	HSS-Ti	HSS-Co	HSS-Mo
Tool diameter (mm)	7	7	7
Tool length (mm)	109.45	109.45	109.45
Helix angle (°)	65	65	65
Helix length (mm)	72.61	72.61	72.61
Chisel length (mm)	1.19	0.31	1.23
Rake angle (°)	8.5	5.6	9.5
Cheap angle (°)	58.85	56	63
Head angle (°)	123	137	114

**Table 3:** Machining parameters.

Parameter	Value
Cutting speed	25 m/min (1137 RPM)
Feed rate	50 mm/min (0.44 mm/rev)
Tool overhang length	73.52 mm

**Table 4:** Design of Experiment.

Test Number	Feed Rate Mode	Tool Material	Pre-Center Drill Hole
1	Continuous	HSS-Ti coat	No
2	G82	HSS-Ti coat	No
3	G83	HSS-Ti coat	No
4	Continuous	HSS-Co	No
5	G82	HSS-Co	No
6	G83	HSS-Co	No
7	Continuous	HSS-Mo	No
8	G82	HSS-Mo	No
9	G83	HSS-Mo	No
10	Continuous	HSS-Ti coat	Yes
11	G82	HSS-Ti coat	Yes
12	G83	HSS-Ti coat	Yes
13	Continuous	HSS-Co	Yes
14	G82	HSS-Co	Yes
15	G83	HSS-Co	Yes
16	Continuous	HSS-Mo	Yes
17	G82	HSS-Mo	Yes
18	G83	HSS-Mo	Yes
